# Sickle Cell Disease in the Post Genomic Era: A Monogenic Disease with a Polygenic Phenotype

**Published:** 2009-07-30

**Authors:** A Driss, KO Asare, JM Hibbert, BE Gee, TV Adamkiewicz, JK Stiles

**Affiliations:** 1 Department of Microbiology, Biochemistry and Immunology, Morehouse School of Medicine, Atlanta, Georgia, USA.; 2 Department of Clinical Pediatrics, Morehouse School of Medicine, Atlanta, Georgia, USA.; 3 Department of Family Medicine, Morehouse School of Medicine, Atlanta, Georgia, USA.

**Keywords:** drepanocytose, genomics, hemoglobin, HbS, hemoglobinopathies, RBC

## Abstract

More than half a century after the discovery of the molecular basis of Sickle Cell Disease (SCD), the causes of the phenotypic heterogeneity of the disease remain unclear. This heterogeneity manifests with different clinical outcomes such as stroke, vaso-occlusive episodes, acute chest syndrome, avascular necrosis, leg ulcers, priapism and retinopathy. These outcomes cannot be explained by the single mutation in the beta-globin gene alone but may be attributed to genetic modifiers and environmental effects. Recent advances in the post human genome sequence era have opened the door for the identification of novel genetic modifiers in SCD. Studies are showing that phenotypes of SCD seem to be modulated by polymorphisms in genes that are involved in inflammation, cell–cell interaction and modulators of oxidant injury and nitric oxide biology. The discovery of genes implicated in different phenotypes will help understanding of the physiopathology of the disease and aid in establishing targeted cures. However, caution is needed in asserting that genetic modifiers are the cause of all SCD phenotypes, because there are other factors such as genetic background of the population, environmental components, socio-economics and psychology that can play significant roles in the clinical heterogeneity.

## Introduction

Hemoglobinopathies are the world’s most widespread genetic diseases.^1^ The high frequency of hemoglobinopathies globally is of great concern to public health managers of various nations.^2^ Among the hemoglobinopathies, sickle cell disease is a genetic disorder in which the beta-chain of the human hemoglobin (Hb) gene is mutated, leading to an abnormal Hb. This mutation causes red blood cells (RBCs) to acquire a sickle shape under conditions of hypoxia, resulting in a very large range of phenotypes such as anemia, cell adhesion, vaso-occlusion, severe pain, stroke and organ failure. For the past several years, genetic studies and single-nucleotide polymorphisms (SNPs) genotyping on patients with various phenotypes showed involvements of SNPs of different genes.^3^,^4^ To date, over 100 SNPs that might be associated with specific phenotypes have been shown to be significantly involved in different genes that act as inflammatory mediators, modulators of oxidant injury, Nitric Oxide (NO) biology, vaso-regulatory molecules and cell adhesion factors. These studies show that SNPs in genes implicated in the transforming growth factor-beta/bone morphogenetic protein (TGF-beta/BMP) pathways and a few other genes such as Klotho gene (KL) are associated with several phenotypes of SCD.

Experiments showed that decreased NO bioavailability, due to scavenging of NO by cell free hemoglobin, a product of the hemolytic process associated with SCD, played a significant role in the vascular patho-biology of SCD.^5^ Thus, the emerging views of the pathogenesis of many complications of SCD involve complex interactions between sickle reticulocytes, neutrophils, monocytes, and the endothelium. It therefore follows that many factors that are also important in the pathways leading to inflammation, cellular adhesion, NO metabolism, vascular reactivity and coagulation are important in the patho-physiology of the disease and that variations in the expression of molecules in these pathways contribute to the heterogeneity of SCD.

The elucidation of genetic variants and mechanisms responsible for the phenotypic variability in SCD will have important clinical implications for genetic counseling and clinical management. Targeted follow up of patients and utilization of individual therapeutic strategies and preventive cures will produce better outcomes for patients with SCD.

In this review, we summarize some of the recent genomic findings focused on non globin genetic modifiers, shown to be potentially implicated in different clinical outcomes of SCD.

### The beta-globin gene and the origin of sickle cell hemoglobin (HbS)

The main Hb function is to transport oxygen to tissues. Adult Hb is composed of a major HbA (alpha2beta2) and a minor HbA2 (alpha2delta2), with traces of fetal HbF (alpha2gamma2). Each of the different globin chains is controlled by distinct genes; two genes exist for the alpha and gamma chains and one for each of the other chains.^2^,^6^ HbS is caused by a single amino acid substitution of Glutamic Acid replaced by Valine at the sixth position of the beta-globin chain. This is due to a single nucleotide substitution, GAG —> GTG in codon 6 of the beta-globin gene on chromosome 11p15.5.^7^ This mutation alters the stability of the Hb and leads to the clinical disorder. The homozygous state of the sickle cell gene mutation (HbSS) results in sickle cell anemia (SCA). The replacement of the Glutamic Acid by a Valine causes Hb polymerization induced by deoxygenation.^8^ As the deoxy-HbS polymerizes and fibers align, the erythrocyte is transformed into a “sickle” shape. These deformed and rigid erythrocytes can obstruct normal blood flow in micro-circulation and thereby induce ischemia in tissues distal to the vascular blockage, which is the basis for many SCD complications.^8^,^9^ Thus, sickling is the direct consequence of the presence of HbS, but pleiotropic effects that follow depict the clinical outcome of the disease.

Since the clinical phenotypes of homozygous HbS are extremely variable, it is now clear that even if SCA is a monogenic disorder, at the phenotypic level it is most likely to be a multigenic disease. Among risk factors for early mortality are stroke, painful crisis and infection.^10^ There are many factors that might influence the outcome of these clinical presentations. These factors include, but are not limited to, environmental, psychological, cultural, and the socio-economical. However, the epigenetic mechanisms seem to play a primordial role in determining different SCD phenotypes.

### Modulation of SCD by other disorders

Genetics is a major determinant of childhood survival in malaria endemic countries. The past few years have seen significant progress towards showing some of the best-known malaria resistance genes that determine the structure or function of RBCs, such as Gerbich blood group antigen negativity, polymorphisms of the complement receptor genes (most notably CR1), Southeast Asian ovalocytosis, pyruvate kinase deficiency; HbE, the sickle cell trait (SCT) and alpha-thalassemia.^11^ Despite conclusive evidence that these defects of the Hb molecule protect against severe and fatal *Plasmodium falciparum* malaria,^12^ the mechanisms underlying this protection are poorly understood.

Alpha-thalassemia is due to mutations of the alpha-globin genes (chromosome 16pter-p13.3) and it has been shown that the presence of alpha-thalassemia has a protective role against *Plasmodium falciparum* malaria infection. This could explain its high gene frequency in geographic malaria-endemic regions. However, several patients with SCA have coincidental alpha-thalassemia and the presence of both SCA and alpha-thalassemia mutations seems to act as a negative epistatic factor.^13^

Alpha-thalassemia reduces the concentration of HbS and therefore of HbS polymerization. Thus it is expected that this will prevent vaso-occlusive events that are consequences of hemolysis, including stroke, leg ulcer, priapism, and pulmonary hypertension. Complications more dependent on blood viscosity, such as painful episodes, acute chest syndrome (ACS) and avascular necrosis will usually be more prevalent when alpha-thalassemia coexists with SCD mutation.^14^,^15^ This is explained by patients with homozygous alpha-thalassemia and SCD having slightly lower levels of HbF than the non-thalassemic sickle cell patients. Preferential survival of F cells, a subpopulation of erythrocytes, occurs in SCA, with or without alpha-thalassemia, and the slight difference in HbF levels appears to reflect differences in numbers of circulating F cells. Thus, the change in the erythrocyte density profile in SCD with coexisting alpha-thalassemia, could explain the change in blood viscosity and the hematological improvement.^16^

Glucose-6-phosphate dehydrogenase (G6PD) deficiency (Chromosome Xq28) is commonly found in HbS populations. Although this deficiency does not appear to have a direct effect on the SCD phenotype,^17^ there are case reports of more severe hemolysis in patients with SCD and G6PD deficiency.^18^ Similarly, coinheritance of SCD and pyruvate kinase (Chromosome 1q21) deficiency can cause painful crisis,^19^ and co-inheritance of sherocytosis may cause recurrent acute splenic sequestration crisis.^20^,^21^ All these examples highlight the complexity of gene interactions.

### Phenotype outcomes and potential modifier gene polymorphisms

The consequences of the sickle mutation and its downstream effects are clearly variable. Complications due to chronic hemolytic anemia, episodic vaso-occlusion with resultant painful episodes and chronic organ damage lead to very variable phenotypes of SCD. It is very difficult to determine the exact factors mediating the severity of the disease. It seems at least that all hematologists agree that they can definitely define the very mild or the “asymptomatic” patients as an obvious phenotype.^22^–^24^ Different authors have reported SCD as an inflammatory disease with endothelium involvement.^25^ Other studies have implicated the NO bio-availability, associated with the scavenging of NO by cell free Hb (product of hemolysis), in the vascular patho-biology of SCD.^5^

It is critical to carefully characterize phenotypes in order to study complex gene interactions. Studies of sickle cell patients from different populations will very likely yield important information due to the differences in genetic backgrounds of these populations and potential implications on the disease phenotype. In the past few years, many centers have focused on the study of genetic modifiers of SCD. Selected findings are summarized here. Table 1 reviews a list of SNPs reported to be significantly associated with different phenotypes of SCD.

#### Fetal Hb and hydroxyurea response

HbF concentration is high at birth and declines slowly during the first three decades of life and the final level in adults is variable. It has been well established that in most cases, HbF has an ameliorative effect on SCD patients because the gamma-chains of HbF do not polymerize with HbS. This potential anti-sickling effect resulted in the development of a major therapeutic product for amelioration of SCD effects. To date, the only drug that is therapeutically used in this way is the S-phase specific chemotherapeutic agent, hydroxyurea (HU) which increases HbF production.^26^

Among SCA patients, HbF concentrations vary from 0.1% to 30% with an average of about 8%. Many laboratories have made efforts to elucidate the reasons behind differences in HbF levels between patients.^27^–^29^ Understanding the mechanisms controlling expression of HbF (gamma-globin gene) may help in the design of better therapeutic strategies in the cure of SCA severity. Unfortunately, some inconsistencies have been reported in patients with very severe disease that have an HbF level of nearly 20% and in some older SCA patients that often have very low levels of HbF.^24^,^30^,^31^ Moreover, the different effect of HbF on the clinical course and the differences in response to HU treatment among patients with similar HbF levels may be related to heterogeneity in the cellular distribution of HbF or to the effect of other modifying genes.^29^,^32^

Several genetic determinants have been shown to contribute to the heterogeneity of baseline HbF levels in SCD. Haplotypes of the beta-globin gene cluster are among the most extensively studied.^33^ High HbF levels are associated with Senegal and Asian-Indian haplotypes, which confer a general milder clinical and hematological phenotype compared with the other African haplotypes (Benin, Bantu and Cameroon). Individuals with Bantu haplotypes have the lowest HbF level and the most severe phenotype.^34^ Individuals with the Benin haplotype usually have intermediate features.^34^ The common feature of the Senegal and Asian-Indian haplotype is the presence of a C->T polymorphism in the promoter of the G-gamma-gene [-158(C->T)] detectable with the *XmnI* restriction enzyme.^35^–^37^ Other studies suggested that the beta-globin gene cluster haplotype, independently of the HbF levels, is correlated with survival of SCA patients treated with HU.^38^ Further research is necessary to understand how that occurs. Modifier genes could be interacting with the beta-globin-like cluster. HbS haplotypes and variations in cis-acting elements associated with different haplotypes are only partially responsible for the variation seen in HbF levels among SCD patients.^39^–^41^ This has prompted the search for transacting regulatory elements controlling HbF levels. Among these elements was cited a quantitative trait locus (QTL) on chromosome Xp22 that was associated with the F cell number production.^39^–^41^ Together with the −158 (C->T) polymorphism in the G-gamma promoter, this locus is estimated to account for half of the variation of HbF level in SCD.^3^ Other QTLs associated with HbF level have been described on chromosome 6q22.3–23.2 in a single extended Asian-Indian family.^29^,^42^,^43^ Another QTL on chromosome 8q was described in an Asian family with beta-thalassemia. Beta-thalassemia is due to different mutations on the beta-globin gene and the severity of the disease depends on the nature of the mutation. This QTL seems to interact with the −158 (C->T) polymorphism.^44^,^45^ Some studies showed the presence of SNPs in intronic regions of genes in the 6q QTL (Table 1). SNPs in several genes were found to be associated with the HbF levels suggesting that these genes are regulatory elements and modulators of HbF.^23^ These genes are phosphodiesterase 7B (PDE7B), microtubule-associated protein 7 (MAP7), mitogen-activated protein kinase kinase kinase 5 (MAP3K5), BCL2-associated transcription factor 1 (BTF) and peroxisomal biogenesis factor 7 (PEX7).^29^ They are also associated with the p38-MAPK pathway with the activation of gamma-globin gene expression by histone deacetylase inhibitors in K562 cells. Another interesting study^46^ screened two panels of 824 and 1,217 individuals of twin pairs from Northern European origin. They selectively chose v-myb myeloblastosis viral oncogene homolog (MYB) and HBS1-like (HBS1L) candidate genes in the 6q23 QTL interval. They identified three linkage disequilibrium (LD) blocks, which span a nearly contiguous segment of 79 kb long, starting 188 bp upstream from HBS1L exon 1 and ending 45 kb upstream of MYB. Among the 12 markers exhibiting the strongest evidence of association, one, rs52090909, is located in the 5′ UTR of exon 1a of HBS1L. The other strongly associated markers are either in intron 1a (rs9376090, rs9399137, rs9402685 and rs11759553), or directly upstream of the 5’ UTR of HBS1L exon 1a (rs4895440, rs4895441, rs9376092, rs9389269, rs9402686, rs11154792 and rs9483788). A test of linkage in the European dizygotic twins showed that the 6q23 QTL was completely accounted for by the markers in the trait-associated blocks. Based on measured haplotype analysis, they estimate that 17.6% of the trait variance is attributed to the markers in the three HBS1L-MYB blocks. An additional 11.6% of the trait variance is influenced by the *XmnI* variant on chromosome 11. As the overall heritability of the cellular HbF trait in Europeans is 89%,^47^ it is suggested that additional genetic or other familial factors contribute substantially (residual heritability = 59.8%) to the trait variation. They suggest that genetic variants that are associated with high F cell levels are also strongly correlated with increased expression of HBS1L in cultured erythroid cells.

Studies in 137 SCA patients treated with HU showed an association between SNPs and the change in HbF level after 2 years of treatment with HU. Using a candidate gene approach, they found SNPs in TOX (thymocyte selection-associated high mobility group box) gene within the 8q12.1 linkage peaks, and SNPs within the 6q22.33 region on mitogen-activated protein kinase kinase kinase 5 (MAP3K5) gene. They reported SNPs in other genes such as hydroxyacid oxidase 2 (HAO2), nitric oxide synthase 1 (NOS1), fms-related tyrosine kinase 1 (FLT1), arginase, type II (ARG2), nitric oxide synthase 2A (NOS2A) and phosphodiesterase 7B (PDE7B) that were associated with the HbF response to HU.^48^

The interactions of NO with Hb result in the formation of a vast biological sink of NO activity that modulates microvascular tone throughout the body. NO reacts with oxygenated Hb and reduced Hb to produce nitrate plus met-Hb and iron-nitrosyl-Hb, respectively; NO has several mechanisms of action, but it appears to play a major role in both the regulation of vascular muscle tone at the cellular level as well as in platelet aggregation (clumping).^49^ In SCD, especially in SCA disease, the rapid release of cell-free Hb may exceed the normal clearance mechanisms such as by binding to haptoglobin, and thus the excess free Hb consumes NO and impairs its regulatory role.^5^

**Figure 1:**
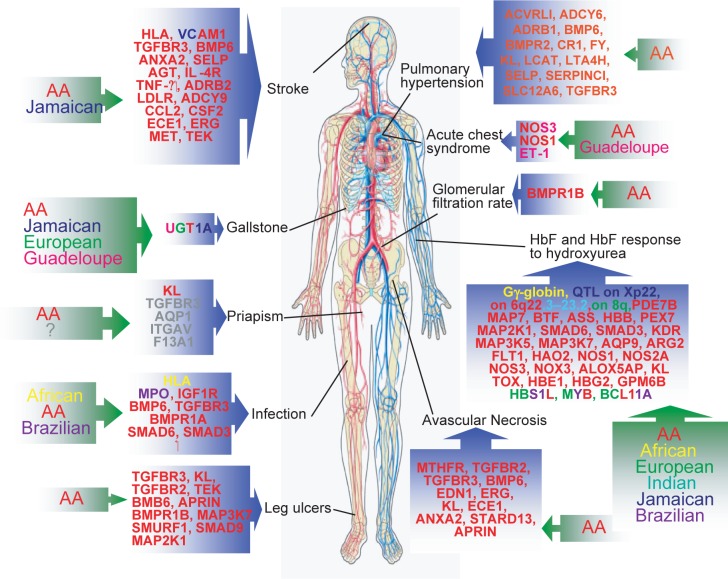
Schematics of genes where SNPs were reported to be significantly implicated on different phenotypes of SCD and on which population it was described. (AA= African American).

Using a newly developed Bayesian modeling approach, the same team used two independent groups of patients, one group of 1,518 adults and children from the Cooperative Study of Sickle Cell Disease (CSSCD), and validated the results in a second independent group of 211 adults from the Multicenter Study of Hydroxyurea (MSH) in SCA.^23^ The study screened about 850 SNPs in 320 candidate genes. They found SNPs in the aquaporin 9 (AQP9), mitogen-activated protein kinase kinase 1 (MAP2K1), SMAD family member 3 (SMAD3), kinase insert domain receptor (KDR), mitogen-activated protein kinase kinase kinase 7 (MAP3K7), NADPH oxidase 3 (NOX3), argininosuccinate synthetase 1 (ASS), NOS1, arachidonate 5-lipoxygenase-activating protein (ALOX5AP), nitric oxide synthase 3 (NOS3), KL and SMAD family member 6 (SMAD6) (15q21.22) suggesting that these genes are regulatory elements in this region and novel modulators of HbF. The SNP rs1867380 in AQP9 is a functional SNP with an amino acid change located in the last exon of the gene. The minimum allele frequency observed for the minor allele A is 22% in the CSSCD set, and 19% in the MSH set which is consistent with 20% estimated in the African American panel of 23 samples reported in dbSNP. HbF levels are slightly lower in subjects who are homozygous for the minor allele A, while heterozygous subjects have a highly variable distribution of HbF. They also reported SNPs in TOX (8q21.1), hemoglobin, epsilon 1 (HBE1) and hemoglobin, gamma G (HBG2) genes, coinciding the beta-globin gene-like cluster (11p15.4) and SNPs in the glycoprotein M6B (GPM6B) gene (Xq22.2 QLT). Other SNPs in different genes have been reported in the CSSCD study only with a difference of association between age groups. Table 1 and Figure 1 summarizes all the SNPs described.^23^

Although the significance of all these data seems attractive, a genome-wide screening using new high throughput technologies is suggested to find how all the polymorphisms in genes regulating HbF expression, HU metabolism and erythroid progenitor proliferation might modulate patient response to HU. Also, it is important to note that, some SNPs and genes found to be associated in the CSSCD population were not found to be associated in the MSH population. Because these two populations were of African American origins, it is possible that some modifier genes may not have originated from an African ancestry. This shows the importance of studying different isolated populations composed of large cohorts of individuals to conduct association studies.

Multiple genes are very likely to affect the response of HU and HbF levels. Their interactions and the predictive values of their polymorphisms will help to elucidate their complex mechanisms.

#### Stroke

Stroke is a devastating complication of SCD, which occurs in about 11% of patients under 20 years of age.^50^,^51^ Among these patients, stroke is predominantly ischemic and results from the involvement of medium sized to large intracranial arteries. Ischemic stroke alone is considered in the general population as a multigenic disorder.^52^–^54^ In many cases it results from gene-gene and gene-environment interactions. In the last few years, genetic determinants have been shown to influence the risk of stroke and many SNPs in different genes have been found to be associated with ischemic stroke.^55^ It was reported that alpha-thalassemia is a protective factor^56^ and increased levels of HbF are associated with reduced risks for other complications.^57^

Studies have shown that increased TCD (Trans Cranial Doppler) velocities of blood flow (>200 cm/sec) in major intracranial arteries, is a predictor of stroke risk in children with SCD.^58^ The value of TCD as a predictor of stroke risk has been validated in the randomized STOP (Stroke Prevention in Sickle Cell Anemia) trial.^58^ As reviewed in Driscoll et al,^59^ genetic association studies in SCA have examined stroke phenotype and have shown the possibility of inherited modulation of this phenotype.^59^ Recent studies have found association of increase stroke risk with several Human leucocyte antigen (HLA) genotypes, especially association of certain phenotypes with distinct subtypes such as large vessel or small vessel stroke.^60^,^61^ HLA DRB1*0301 and *0302 alleles increased the susceptibility of stroke while the DRB1*1501 protected against stroke.^62^ DRB1*0201, in LD with DRB1*0301, was associated with stroke and DQB1*0602, in LD with DRB1*1501, was protective from stroke. Using the CSSCD patient database, HLA genotyping was performed in 36 patients with large vessel stroke and 35 with small vessel stroke.^60^,^62^ A total of 160 patients with a negative magnetic resonance imaging scan served as controls. In the small vessel stroke group, HLA DPB1*0401 was associated with increased stroke risk, whereas DPB1*1701 conferred protection from stroke. The DPB1*0401 allele was associated with susceptibility and DPB1*1701 was associated with a trend toward protection from stroke in the small vessel stroke group. In the large vessel stroke subgroup, HLA-A*0102 and *2612 conferred susceptibility, whereas the allele *3301 protected from stroke. These results suggest that specific HLA alleles influence stroke risk and appear to contribute differently to small vessel and large vessel stroke subtypes. Therefore, different pathologic processes may be involved in the development of stroke in children with SCA.^60^ Separation of ischemic stroke into subtypes based on presumed mechanisms may help clarify the contribution of HLA to stroke risk in SCA. These results need to be confirmed in studies with a larger number of patients, and using different populations’ if possible.

In a study of seven candidate genes in 42 children with large vessel cerebral artery stenosis, SNPs in the transforming growth factor-β receptor 3 (TGFBR3) and adenine cyclase 9 (ADCY9) were associated with this phenotype, when compared with 71 controls.^3^,^59^

Other studies, investigating candidate genes in about 230 children from the CSSCD population found associations of stroke with several genes including interleukin-4 receptor (IL-4R) S503P, tumor necrosis factor super-family, member 2 (TNFalpha) −308G and adrenergic beta-2 receptor (ADRB2) Q27E polymorphisms with large vessel stroke and vascular cell adhesion molecule 1 (VCAM1) −1594T>C, low density lipoprotein receptor (LDLR) *NcoI* polymorphisms with small vessel stroke.^61^,^63^ According to this study the combination of TNFalpha-308 GG homozygosity and IL-4R S503P polymorphism conferred a particularly strong risk for large vessel stroke. Another study reported polymorphism VCAM1 G1238C as protective against symptomatic stroke.^64^ Independent studies from the STOP population confirmed the association of large vessel stroke in SCD with both TNFalpha −308G and IL-4R S503P polymorphisms^63^ as well as the association of VCAM1 G1238C.^65^

Another study^66^ analyzed 108 SNPs in 39 candidate genes in 1,398 individuals from the CSSCD African American population with SCD. Thirty-one SNPs in 12 genes were found to interact with HbF to modulate the risk of stroke. This network of interactions includes three genes in the TGF-beta pathway and selectin P (SELP), which is associated with stroke in the general population.^67^ The researchers used the Bayesian networks modeling to capture the relationship between genotypes and phenotypes that can be used to compute the probability that a new individual with a particular genotype, will have the phenotype of interest.^68^ The network dissects the genetic basis of stroke into 11 genes whose variants have a direct effect on the disease that is modulated by HbF levels, and 9 genes whose variants are indirectly associated with stroke. For example, the cluster of five SNPs in endothelin 1 (EDN1) is associated with SNPs in annexin A2 (ANXA2) and bone morphogenetic protein 6 (BMP6). Both EDN1 and BMP6 are on chromosome 6 (at 6p24.1 and 6p24.3, respectively), and their association suggests that this chromosomal region may be associated with an increased risk of stroke. ANXA2 has a regulatory role in cell surface plasmin generation; EDN1 might be a potent vaso-constrictor and mitogen secreted in response to hypoxia, supporting the hypothesis that EDN1 antagonists may be useful in the prevention and treatment of sickle vaso-occlusive crises. They suggest that these model variants in BMP6 are the strongest risk factors, whereas variants in EDN1 are associated with stroke through BMP6 and ANXA2 but are not as relevant for the risk prediction. Stroke is also directly associated with variants in TGFBR3 and indirectly associated with variants in transforming growth factor, beta receptor II (TGFBR2), which have essential, non-redundant roles in TGF-betạ signaling. BMP6 is part of the TGF-beta super-family, and the simultaneous association of three genes with functional roles in TGF-betạ signaling suggests that this pathway might be involved with increased risk of stroke. This conjecture is further supported by the association of stroke with colony stimulating factor 2 (CSF2), a protein necessary for the survival, proliferation and differentiation of leukocyte progenitors. Using this method, they can compute the probability distribution of the phenotype (stroke) given the genotype of any SNP and, conversely, compute the conditional distribution of any genotype given values of other variables in the network. The overall predictive accuracy reported is 98.2%.^66^ In this way, the model is able to describe the determinant effects of genetic variants on stroke, to predict the odds for stroke of new individuals given their genotypes and to find the most probable combination of genetic variants leading to stroke. This method is very promising in that it can be used to find relationships between genotypes and phenotypes of all the SCD symptoms. Other genes involved according to this study are ADCY9, chemokine (C-C motif) ligand 2 (CCL2), endothelin converting enzyme 1 (ECE1), v-ets erythroblastosis virus E26 oncogene homolog (ERG), hepatocyte growth factor receptor (MET) and TEK tyrosine kinase (TEK).

Other studies attempted to associate stroke risk with SNPs in the angiotensinogen (AGT), cystathionine-beta-synthase (CBS), cholesteryl ester transfer protein (CETP), integrin, beta 3 (ITGB3), apolipoprotein C-III (APOC3), 5,10-methylenetetrahydrofolate reductase (MTHFR), serpin peptidase inhibitor, clade E (PAI1), intercellular adhesion molecule 1 (ICAM1) and thrombospondin receptor (CD36) genes,^64^,^69^ but due to the few patients studied, replication of these results in a larger patient sample is necessary.

To further define the genetic basis of stroke, the association of SNPs in candidate genes of different functional classes with the likelihood of having a stroke was examined. A total of 113 patients with SCA and a confirmed history of, or incident complete, non-hemorrhagic stroke, documented by imaging studies were compared with 493 control patients. Polymorphisms in four candidate genes, KL, TGFBR3, ANXA2 and BMP6, were associated with stroke.^70^ These genes play roles in the TGF-beta/BMP pathway, cell adhesion and NO biology. KL (13q12) encodes a membrane protein and regulates many vascular functions including vascular endothelial growth factor expression and NO release by the endothelium.

No association with cerebro-vascular disease was found when multiple coagulation factors were measured in children transfused for stroke, at risk for stroke and in untransfused controls.^71^–^73^ Polymorphisms in low-affinity Fc leucocyte receptors were not associated with stroke.^64^ Hypoxia-induced cellular activation and release of adhesive and inflammatory mediators might be related to stroke and other vaso-occlusive complications, as suggested by a study of cell adhesion molecules in children with mild sleep hypoxia who had a higher vaso-occlusive episodes and increased markers of cell adhesion and activation.^74^

To examine the interaction among genes and their variant SNPs and to develop a prognostic model for stroke in SCA, a Bayesian network was developed to analyze 235 SNPs in 80 candidate genes in 1398 unrelated subjects with SCA. SNPs on 11 genes and four clinical variables, including alpha-thalassemia and HbF, interacted in a complex network of dependency to modulate the risk of stroke. This network of interactions included three genes, BMP6, TGFBR2, TGFBR3 with a functional role in the TGF-beta pathway and one gene (SELP) associated with stroke in the general population. The model was validated in a different population by predicting the occurrence of stroke in 114 unrelated individuals with 98% accuracy, predicting the correct outcome for all seven stroke subjects, and for 105 of 107 non-stroke subjects. This gave a 100% true positive rate and 98. 14% true negative rate, and an overall predictive accuracy of 98%.^66^ As traditional analytical methods are often inadequate for the discovery of the genetic basis of complex traits in large association studies, Bayesian networks are emerging as a promising approach. The predictive accuracy of this stroke model is a step toward the development of prognostic tests which will allow a better identification of patients at risk for stroke. The presence among the risk factors of genes already associated with stroke in the general population, such as SELP, suggests that some genetic factors predisposing to stroke may be shared by both SCA patients and stroke victims in general.

#### Avascular necrosis/Osteonecrosis

In the CSSCD study it was shown that alpha-thalassemia, age, high hematocrit and frequent vaso-occlusive events, are risk factors for the development of avascular necrosis (AVN) of the femoral head in SCD.^75^ Kutlar et al investigated the frequency of the MTHFR C677T gene polymorphism in the sickle cell population as it is associated with elevated serum homocysteine levels and resultant vascular complications in relation with AVN. They discovered that MTHFR C677T was present in 16% of the sickle cell patients. There was a strong association of the presence of MTHFR C677T with AVN in 35.6% of the AVN patients having the MTHFR polymorphism as opposed to only 12.9% of sickle cell patients without AVN.^76^ This association has been confirmed in a Brazilian population,^77^ however, this was not confirmed in the high Hb F population of Kuwaiti sickle cell patients, suggesting that different genetic factors may be operative in different populations.^78^ Some other smaller studies in different patient populations have also failed to show an association between this MTHFR polymorphism and the risk of AVN in SCD.^72^,^79^,^80^ A recent study^81^ analyzed 442 patients with AVN and 455 SCD controls from the CSSCD cohort. They studied SNPs in 66 candidate genes and found significant association of AVN with seven SNPs in BMP6, TGFBR2, TGFBR3, EDN1, ERG, KL, ECE1, ANXA2, StAR-related lipid transfer domain containing 13 (STARD13) and PDS5, regulator of cohesion maintenance, homolog B (APRIN) genes. The precise mechanism(s) whereby variation in these genes causes AVN is not yet understood.

#### Acute chest syndrome

Increased susceptibility to ACS has been associated with the T-786C SNP in the NOS3 gene in females.^82^ Gender specific disease modifications in the endothelial NOS3 have been proposed as explanation for these modifications.^83^ It is suggested that the differences between sexes could possibly be explained by modulation of NOS3 activity through circulating estrogen, resulting in differences of NO formation in pulmonary vascular endothelium between females and males. Exhaled NO is significantly lower in healthy females compared with males, and in the case of relative NO deficiency, such as in SCD and CF, NOS3 variants associated with altered response to female hormones may then be relevant for the pathophysiology of the disease.^83^ Low exhaled levels of NO were also seen in patients with ACS compared with controls, and this was associated with the number of AAT repeats in intron 20 of NOS1.^84^ Another recent study shows that endothelin 1 (ET-1) T8002C and NOS3 C-786 alleles are associated with both an increased and a decreased risk of ACS in SCA patients.^85^ It was also proposed that Secretory phospholipase A(2) (sPLA2) may be related to the severity of the ACS and capable of predicting its onset.^62^ sPLA2, is found in low concentration in normal plasma, however, its levels are increased in response to inflammation and were found to be very high in the ACS.^86^

#### Priapism

Priapism, a persistent, usually painful, erection that lasts for more than four hours and occurs without sexual stimulation, occurs in 30%–45% of male patients with SCD. The possible influence of genetic risk factors on the incidence of priapism is not well understood. A study made on 44 candidate genes, in 148 patients with SCA and a confirmed history of priapism, versus 529 control SCA patients who never developed priapism, revealed a polymorphic association with the KL gene.^87^ Another recent study examined genetic polymorphisms in 199 unrelated, adult (>18 years) male patients with HbSS and HbS/beta (0)-thalassemia, 83 (42%) with a reported history of priapism. Candidate genes for association with priapism were identified based on their involvement in adhesion, coagulation, inflammation and cell signaling. They also examined genes involved in nitric oxide biology (NOS2, NOS3, SLC4A1), as well as polymorphisms in the KL gene. They reported strong evidence of association found for SNPs in the TGFBR3, aquaporin 1 (AQP1), integrin, alpha V (ITGAV), and the coagulation factor XIII, A1 polypeptide (F13A1). Associations with TGFBR3, AQP1, and ITGAV remained significant after adjusting for multiple testing, using the Benjamini-Hochberg procedure. These data suggest that genes involved in the TGF-β pathway, coagulation, cell adhesion and cell hydration pathways may be important in risk for priapism.^88^ In this study however, associations with the SNPs in the KL gene as reported before, were not significant.

#### Leg ulcer

Cutaneous leg ulcers are common in SCA. In the United States, 2.5% of patients with all common genotypes of SCD have leg ulcers.^89^ In Jamaica, >40% of patients^90^ and between 1.5% and 13.5% of SCD patients in Africa were reported to have leg ulcers.^91^–^93^

In a study made on the CSSCD cohort, and after screening 215 SNPs in more than 100 candidate genes, associations were found with SNPs in KL, TEK and several genes in the TGF-beta/BMP signaling pathway by genotypic association analyses. KL directly or indirectly promotes endothelial NO production and the TEK receptor tyrosine kinase is involved in angiogenesis. The TGF-beta/BMP signaling pathway modulates wound healing and angiogenesis, among its other functions. They suggest that hemolysis-driven phenotypes, such as leg ulcers, could be improved by agents that reduce sickle erythrocyte density or increase NO bioavailability.^94^ Among the genes reported to be significantly associated are: TGFBR3, TGFBR2, BMP6, TEK, KL, APRIN, bone morphogenetic protein receptor, type IB (BMPR1B), SMAD specific E3 ubiquitin protein ligase 1 (SMURF1), MAP3K7, SMAD family member 9 (SMAD9) and MAP2K1.^94^

#### Gallstones, cholelithiasis and bilirubin levels

Twin and family linkage studies suggested a genetic component to the development of gallstones.^95^–^97^ Pigment stones are the predominant variety in SCD due to chronic hemolysis. Homozygosity for the promoter 7(TA) repeat in the UDP glucuronosyltransferase 1 family, polypeptide A cluster (UGT1A) gene promoter has been associated with unconjugated hyperbilirubinaemia and Gilbert syndrome.^98^,^99^ The normal UGT1A promoter contains an A(TA)nTAA nucleotide-sequence motif with 6 (TA) dinucleotide repeats. A shorter 5(TA) repeat and longer 8(TA) allele have been described in persons of African descent^100^ but without clinical correlation. UGT1A promoter polymorphisms act as an important genetic modifier of hepatobiliary disease in SCA, influencing baseline bilirubin levels and the incidence of cholecystectomy.^101^ This suggests that symptomatic cholelithiasis is more common in carriers of this genotype.^101^,^102^ When treated with HU, children with the 6/6 UGT1A genotype had normal bilirubin levels compared with individuals with the 6/7 or 7/7 genotypes. A recent study suggested that the 7/7 and 7/8 genotypes were risk factors for symptomatic gallstones only in older subjects with SCD.^103^

Carriers of the 7/7 genotype had bilirubin levels greater than 51,3 μmol/l despite full-dose hydroxyurea therapy,^104^ suggesting that this polymorphism may influence the ability of hydroxyurea to prevent gallstone formation. In this study, they analyzed the effect of the UGT1A genotype on the response to HU therapy, which decreases hemolysis in children with SCA with significant increase in Hb concentration, decreases in reticulocytes and lower serum total bilirubin.^105^ They analyzed the reduction in serum bilirubin that occurs with decreased hemolysis in association with HU therapy.^104^ In a large cohort of children with SCA, from the Duke Pediatric Sickle Cell program, taking HU therapy at the maximum tolerated dose demonstrated significant reductions in hemolysis independent of UGT1A promoter polymorphism genotype, but there were no HU-related decreases in serum bilirubin levels. Children with the wild-type 6/6 UGT1A genotype demonstrated normalized bilirubin levels with HU therapy, but children with the heterozygous 6/7 or abnormal 7/7 genotypes did not. Children with the abnormal 7/7 genotype, which confers the phenotype of Gilbert syndrome, had bilirubin levels greater than 3 mg/dL despite full-dose HU therapy. These data indicate that the UGT1A promoter polymorphism is a powerful non-globin genetic modifier in SCA that influences serum bilirubin both at baseline and on HU therapy. UGT1A promoter polymorphisms may therefore influence the ability of HU to prevent gallstone formation in patients with SCA.^104^ Similar findings were confirmed in various cohort studies including Jamaica,^103^ Guadeloupe^106^ and Greece^107^ which makes this modifier gene consistent with different populations.

#### Infection and bacteremia

Infection and bacteremia are common in SCD and severe bacterial infections are the major causes of morbidity and mortality in SCA. A recent study on the CSSCD cohort, showed significant associations with SNPs in insulin-like growth factor 1 receptor (IGF1R) and genes of the TGF-beta/BMP pathway (BMP6, TGFBR3, bone morphogenetic protein receptor, type IA (BMPR1A), SMAD6 and SMAD3) suggesting that both could play important roles in immune function in SCA and their polymorphisms may help identify a “bacteremia-prone” phenotype.^108^

Another study on 144 sub-Saharan African SCD patients, 73 of whom had at least one severe bacterial infection history and 71 had none, showed a bi-allelic polymorphism (Arg107Gly) distribution of a human leukocyte antigen–E (HLA-E) locus. The HLA-E*0101/E*0101 genotype was more frequent among the group with infections than their counterparts (47% vs. 21%). This genetic association is of relevance for the involvement of HLA-E molecules in host response to pathogens.^109^,^110^ Another study involving a small group of subjects from a Brazilian cohort with SCD identified a G463A polymorphism in the myeloperoxidase (MPO) gene.^111^

#### Albuminuria and glomerular filtration rate

The glomerular filtration rate (GFR) in SCA is supra-normal in childhood but falls quickly with age, often resulting in renal failure. The renal failure is common among older patients with homozygous SCA and contributes to death of adult patients.^112^ The risk factors underlying these observations are unclear. Studies have correlated the high GFR with macroalbuminuria and proteinemia.^113^,^114^ In a study of 1,140 patients with SCA, SNPs of 70 candidate genes of the TGF-β/BMP pathway were screened for association with GFR. They found 4 SNPs (rs2240036, rs4145993, rs17022863, rs1434549) in BMPR1B, to be significantly associated. Three haplotypes in this gene were also associated with GFR. The TGF-beta/BMP pathway has been also associated with the development of diabetic nephropathy, which has some features in common with sickle cell nephropathy. These results suggest that, as with other sub-phenotypes of SCD, renal function may be genetically modulated.^94^

#### Pulmonary hypertension

Genetic studies concerning this phenotype are very limited. One recent study measured with a tricuspid regurgitation jet of >2.5 m/sec, showed evidence of association on genes of the TGF-beta superfamily, including activin A receptor type II–like 1 (*ACVRL1*), bone morphogenetic protein receptor 2 (*BMPR2*), and *BMP6*, the beta-1 adrenergic receptor (*ADRB1*).^115^

Gene polymorphisms have been found associated with eNOS and ACE genes in other diseases dealing with pulmonary hypertension-like asthma and chronic obstructive pulmonary disease^116^,^117^ emphasizing the NO pathway again as contributing candidate genes for severity.

#### Sickle cell adhesion

Sickle (SS) RBCs, unlike unaffected (AA) RBCs, adhere avidly to components of the vascular wall, and this abnormal adhesion is believed to contribute to the painful vaso-occlusive crises that occur in patients with SCA.^118^ It was reported that up-regulation of intracellular cyclic adenosine monophosphate (cAMP)-dependent protein kinase A (PKA) by epinephrine significantly increased sickle but not normal erythrocyte adhesion to both primary and immortalized endothelial cells.^119^ This suggests that adrenergic hormones such as epinephrine may initiate or exacerbate vaso-occlusion and thus contribute to the association of vaso-occlusive events with physiologic stress.^119^ In an other study, the same group showed that the alphavbeta3 integrin is the extra-cellular ligand involved in the adhesion of SS RBCs through the LW blood group antigen glycoprotein (a member of the ICAM subfamily).^120^ They conclude that LW appears to be the SS RBC receptor that mediates binding to at least one endothelial integrin, the alphavbeta3. Furthermore, stress hormones, such as epinephrine, may contribute to vaso-occlusion by activating LW to mediate adhesion at least in part through cAMP/PKA-dependent signaling pathways.

A recent study suggested that polymorphisms in the beta2-adrenergic receptors (ADRB2) and adenylate cyclase 6 (ADCY6) may influence SCD severity through the signalling pathway of RBC adhesion to laminin.^121^ In this study, they found that SNP rs1042713 in ADRB2 and SNP rs3730070 of ADCY6 polymorphisms were associated with elevated adhesion (*P* = 0.037 and *P* = 0.0002 respectively). These two genes are activated by adrenaline and may affect SS RBC adhesion to laminin. They theorize that these two genetic polymorphisms of signalling pathways on SS RBC adhesion may lead to pathophysiological consequences as well as disease variability in SCD. Future investigations are needed to confirm a role for adrenaline-mediated in vivo activation of SS RBC adhesion in vaso-occlusion.

#### Painful episodes

Acute painful episodes are a major complication of SCD and can be predictive of early death in adults. The only genetic modulations known to date about pain in SCA are the protective role of HbF and the deleterious or neutral role of alpha-thalassemia.^122^ A major limitation of the polymorphism study is how painful episodes are defined. The detection of genomic variations leading to painful episodes will be very important for SCD patients. The finding of new candidate genes will open the possibilities to use them as biomarkers to diagnose the severity of the disease.

#### Other phenotypes

Other complications can be associated with genetic predisposition. Although only few studies exist on the risk factors of some of these phenotypes, many of them remain to be studied. Some potential sub-phenotypes that can be considered genetically associated with the disease, and thus needing further genetic association studies are: Mortality, dactylitis, aplastc crisis, acute splenic sequestration, chronic hypersplenism, megaloblastic change, iron and folate deficiency, bone pain, pregnancy complications, osteomyelitis, acute pulmonary sequestration, pulmonary fat embolism, deep jaundice, iris atrophy and eye complications and chronic transfusion complications.^3^,^4^,^123^ All these complications, may or may not be directly or indirectly associated with a gene polymorphism, but further studies should be considered.

#### The “no complication” phenotype

Due to the advances in alternative controls and therapies such as HU treatment, transfusions, prophylaxis and antibiotics to prevent infection, new born screening programs, prevention and gene counseling, and improving of socio-economical features, the survival rate of SCA patients is clearly increasing.^57^,^124^ Determining the severity of the disease is very difficult. Isolating one specific sub-phenotype for genetic association screening requires very careful monitoring of the patient’s complications throughout their life. The only clear phenotype that we can surely characterize is the “no complication” phenotype. Indeed, some patients that carry the SS mutated hemoglobin have very mild or no complication throughout their life. We can imagine that these SS individuals carry a gene polymorphism that “protects” them from anemia and vaso-occlusive events. In many of the studies above, they used these patients as negative controls to find polymorphisms in different sub-phenotypes. Some patients with the SS mutation can survive until their 6th and 7th decade (Serjeant, personal communication).^24^ Further studies on gene polymorphisms in these individuals can reveal interesting findings.

## Discussion

### Differences between populations

The beta-globin gene is located on the short arm of Chromosome 11. Variations in non-coding nucleotides flanking the globin genes have been used to track the origin of the sickle cell mutation and its flow to other geographical regions in the world. Using restriction endonucleases, haplotype blocks of 8 SNPs spanning ∼30,000 bases of the beta-globin locus could be determined and was used to study the ancestral origin of the sickle cell mutation in individuals from different parts of Africa and Asia. In Africa, the HbS gene was associated with four haplotypes representing regions where independent mutations occurred. These haplotypes include the Benin, the Senegal, the Bantu (Central African Republic) and the Cameroon.^36^,^125^ A fifth Asian beta-locus haplotype was reported in Saudi Arabia and India.^126^ In America, Jamaica and Brazil, the African ethnic groups were subject to a considerable admixture of different haplotype groups. Calculations suggest that the HbS-globin gene mutation first appeared approximately 70,000∼150,000 years ago.^127^ As described above in the *Fetal Hb and hydroxyurea response* section, the Senegal and Asian-Indian haplotypes have a general milder clinical and hematological phenotype compared with the other African haplotypes (Benin, Bantu and Cameroon). Individuals with Bantu haplotypes have the most severe phenotype and individuals with the Benin haplotype usually have intermediate features.^34^

### Global frequency and distribution

While the mutation prevalence is the highest in the Mediterranean, Africa and Asia, the migration of the populations from these areas has increased globally.^128^ SCD is now endemic throughout Europe, the Americas and Australia.^1^

Comprehensive control programs in recent years have succeeded in limiting the numbers of new births and prolonging life in affected individuals. Such programs have been successful in a minority of countries and have little global impact. Over 300,000 infants with major syndromes are born every year and the majority dies undiagnosed, untreated or under-treated. Countries may be divided into three general categories according to the services available: A. Endemic Mediterranean countries. In these countries, long-established prevention programs have achieved 80 to 100% prevention through specialized clinics which provide optimum treatment. B. Areas of the developed, industrialized world where prevalence is increasing because of migration. These countries have the means to provide adequate control but have problems in reaching immigrant groups with different cultural background. C. Countries of the developing world where the provision of services is hampered by economic difficulties, other health priorities due to high infant mortality from infectious diseases, and religious/cultural constraints.^129^

#### Cooperative studies

In Table 1 the majority of polymorphism discoveries on sub-phenotypes have been made on the CSSCD cohort. This shows the importance of establishing organized cooperative research centers and new born screening projects that help not only for gene polymorphism discoveries of modifier genes, but also enables us to understand the exact mechanism leading to different phenotypes. To conduct such genetic studies, it is very important to have a large number of patients included in each study. The relevance of the findings strongly depends on the statistical significance of the genetic results. This significance is directly associated with the number of individuals included in the study, which defines the power of the results. Independent studies, using different techniques and approaches (as well as different laboratories) must be done, even if the screening will be conducted on the same population. The findings will be additive and will confirm previous results. Confirmation of modifier gene action will enhance the discovery of a potential cure for that phenotype, not only for SCD but also for other diseases sharing similar phenotypes.

The National Institutes of Health (NIH) has established funds to monitor clinical trials and human genetic studies. However, genetic studies require a large number of patient DNA and clinical information. To collect information on patients and protect the personal privacy of each individual, the institutional review board (IRB), which is an independent committee of physicians, statisticians, community advocates and others, insure that the clinical trial or the genetic studies conducted by the investigators are ethical, and all the rights of the studied participants are protected. In the past however, minorities and populations from developing countries were exploited and abused in several clinical trials which has created resentment and suspicion of researchers among these populations. Regaining the trust of minorities and establishment of collaborative studies with developing countries to participate in large scale genetic screenings remains a problematic and challenging task (US DHHS 1994). Recruiting volunteer participants for medical research is critical to developing medical innovation toward improved patient health. A sophisticated and clear collaborative policy will definitely help regain trust in medical research, the backbone of medical cure.

#### Comparative studies between populations

SCD is one of more than 10,000 human diseases that are caused by defects in single genes. Screening for a diverse group of serious and common disorders in the prenatal setting presents a great challenge.

Although it is well known that 85%–95% of human genetic variation is due to variation among individuals within a population, whereas 5%–15% is attributable to variation among populations,^130^,^131^ it remains unclear whether similar levels of within-versus among-population components of variation will extend to higher-level phenotypes such as gene-expression levels. Understanding patterns of gene-expression variation within and among human populations will provide important insights into the molecular basis of phenotypic diversity and the interpretation of patterns of expression variation in disease. Finding that a single locus has a strong signal in two different populations and in two separate studies, is very important in validating biomarkers for the phenotype. Such results may be combined to maximize the amount of available information that can be extracted from these expensive and laborious experiments.

One example of the importance of comparing the population SNPs is the MTHFR gene described above and its implication in the avascular necrosis. This gene has been found associated with this phenotype in a large number of patients of the CSSCD population but could not be confirmed in the Kuwaiti population.^78^ This suggests that different genetic factors may be operative in different populations. On the other hand the UGT1A gene promoter (TA) repeat polymorphism has been confirmed in different populations, making this gene a potentially reliable biomarker that can be used as a diagnostic predictor.

#### Importance of collecting environmental information including diet and traditions

The differences between populations do not only refer to a pure genetic paradigm, but also refer to geographic, social, dietetic, traditional and socio-economical matter. The outcome of the hemoglobin sickling on patients eating different diet will be different. Even though very few studies has been made on water intake and it’s real patho-physiological influence on the phenotype, doctors advise children with SCA to drink water and fluids and avoid dehydration. Very few studies have been done on the effect of diet on SCD patients. Some studies done in our laboratory, showed correlations between the metabolic demands of increased erythropoiesis and cardiac energy consumption with the excess protein and energy metabolism in children with SCA.^132^ The same group conducted studies using a sickle cell mouse model and showed a direct role of the protein intake on the outcome of the disease.^133^

SCD is a blood disorder. Blood does not only carry oxygen and carbon dioxide, but also has primordial roles in distribution of water, metabolites, energy, hormones and enzymes to different parts of the body. It is logical that the diet, environmental factors and lifestyle in general, as well as psychological and socio-economical factors will definitely influence the outcome of the disease. It is important to note that, when we genetically screen patients for polymorphisms, we take into consideration the environmental factors that could influence the outcome of the disease.

#### Importance of conducting genetic studies in endemic malaria populations

For thousands of years, in sub-Saharan Africa, malaria transmission resulted in natural selection which resulted in preferential killing of healthy and SCA children while individuals with SCT were protected. This resulted in high frequency of SCD patients in malaria endemic regions. The global frequency of hemoglobinopathies shows a remarkably consistent match of hemoglobinopathies with malaria endemic region. The eradication of malaria in some countries, such as in the Mediterranean countries, has contributed to the lower frequency of HbS in these regions. Interestingly, SCD is a genetic disorder while malaria is due to a parasitic infection. It is therefore important to continue the fight to completely eradicate malaria in all the countries.

Susceptibility/resistance to *Plasmodium falciparum* malaria has been correlated with polymorphisms in more than 30 human genes on patients from Africa and south-east Asia and India.^134^

Genome-wide association analysis based on 10,000 single-nucleotide polymorphisms in a Ghanaian population identified several regions which showed evidence for linkage disequilibrium to parasitological and clinical phenotypes. Among them a prominent signal on Chromosome 10p15 obtained with malaria fever episodes^135^ and on Chromosome 5q31–q33.^136^ Several human gene SNPs have been described to be linked with the severity of the malaria such as: CYBB gene,^137^ the gp91 phox subunit of the NADPH oxidase,^137^ the TNF-enhancer and gene for FcgammaRIIa,^134^ IL12B^136^ as well as the haptoglobin haplotype polymorphism.^138^ These studies show a relationship between red blood cell efficiency and the severity of the malaria as observed in the SCA.

#### Benefits and limitations

It is extremely important to utilize new high throughput technologies now available commercially to determine how best to translate such progress into improved patient care. Nowadays, a single DNA chip microarray can be used to screen a whole genome-wide SNPs panel per individual. New technologies have enabled genome-wide association studies to be conducted with hundreds of thousands of genotyped SNPs. Several different first-generation genome-wide panels of SNPs have been commercialized. The total amount of common genetic variation is still unknown; however, the coverage of commercial panels can be evaluated against reference population samples genotyped by the International HapMap project. Less information is available about coverage in samples from other populations. The high cost of these technologies is a major limitation not only for the gene polymorphism studies, but also for the practical exploitation of the biomarker-based therapies. But, despite the high cost, the new technologies became more “per-sample” cost effective and can allow the determination of a whole patient’s genotype in one single experiment. It also gives us the copy number of the gene expression. This advancement of biomedical technologies enables the efficient and reliable screening of patients with different phenotypes and also appears to be the key for establishing biomakers that will allow better diagnosis and treatment of patients that have a susceptibility SNP implicated in the clinical manifestation of the disease. These technologies have been proven to be effective in revealing associations between SNPs and many disease phenotypes, even in non monogenic disorders such as infectious diseases and in different populations including Caucasians, Asians and Africans.^135^,^139^

On the other hand, a recent twin study, introduced a controversy in the paradigm of epistatic genetic control of disease.^140^ This study compared 9 pairs of identical twins of a Jamaican cohort and showed that twins presented similarities in the prevalence and degree of splenomegaly, susceptibility to priapism, and in onset of menarche, but other clinical complications were discordant in prevalence and severity. These findings suggest that physical growth and many hematological characteristics are subject to genetic influences, but that non-genetic factors contribute to the variance in disease manifestations. Thus, in these studies, even though very limited in the sample size, a better understanding of phenotype/genotype correlation in SCD can be acquired.

Coordinating efforts and cooperative studies with different laboratories and hospitals around the world, setting up a common database that is freely shared, and helping developing countries to set up new born screening centers for collecting data will facilitate the search for a cure for SCD.

These kind of genomic investigations are very important for the detection of genomic variations leading to different phenotypes in SCD patients. However, functional studies will be required to correlate the results with the phenotypes. These studies are limited to the extent to which it is a genetic study using new genomic tools and the complexity of genetic interactions and environmental factors require further gene expression confirmations such RNA and protein levels.

The understanding of genomic variations in SCD patients will be key in the use of possible biomarkers for diagnostic of the severity of SCD and that is critical for targeted therapeutics delivery and personalized treatments.

## Figures and Tables

**Table 1:** Review of polymorphisms reported to date to be significantly associated with different SCD phenotypes. (*) = protective.

Phenotype		Gene	Chromosome	GeneID	SNP or allele	*p* value	Population	Ref
**HbF and HbF response to hydroxyurea**		HBG2	11p15.5	3048	**−158 C->T (XmnI)**	N/S	West African	141
		HBB	11p15.5	3043	**rs7482144 (XmnI)**	4 × 10−7	CSSCD	142
		GPM6B	Xp22.2	2824	rs1005589	0.047	CSSCD, MSH	143
					**rs11095629**	0.042	CSSCD, MSH	143
					rs5978663	0.064	CSSCD	143
					rs11095629	0.069	CSSCD	143
					rs7890737	0.07	CSSCD	143
					rs4830513	0.068	CSSCD	143
					rs5979998	0.069	CSSCD	143
					rs6654096	0.067	CSSCD	143
		TOX	8q12.1	9760	rs10504269	0.054	CSSCD, MSH	143
					rs6997859	0.052	CSSCD	143
					rs12155519	0.056	CSSCD, MSH	143
					rs1947178	0.056	CSSCD	143
					rs389349	0.039	CSSCD, MSH	143
					rs4737532	0.058	CSSCD	143
					rs851800	0.045	CSSCD	143
					rs2726599	0.065	CSSCD	143
					rs3109904	0.061	CSSCD	143
					rs7821556	0.061	CSSCD	143
					rs7817609	0.066	CSSCD	143
					rs826730	0.064	CSSCD	143
					rs3779999	0.063	CSSCD	143
					rs1349115	0.066	CSSCD	143
					rs2594953	0.065	CSSCD	143
					rs10283344	0.063	CSSCD	143
					rs12545204	0.066	CSSCD	143
					rs380620	0.062	CSSCD	143
					rs826729	0.045	MSH	48
					rs765587	0.031	MSH	48
					**rs9693712**	0.0098	MSH	48
					rs172652	0.049	MSH	48
					rs380620	0.016	MSH	48
					rs2693430	0.049	MSH	48
					rs12155519	0.037	MSH	48
		HBE1	11p15.5	3046	rs7130110	0.07	CSSCD, MSH	143
					**rs3759070**	0.060	CSSCD	143
		HBG2	11p15.5	3048	**rs7482144**	0.07	CSSCD, MSH	143
		AQP9	15q22.1–q22.2	366	**rs1867380**	0.044	CSSCD, MSH	143
		MAP2K1	15q22.1–q22.33	5604	**rs4489951**	0.051	CSSCD	143
		SMAD6	15q21–q22	4091	**rs1440372**	0.042	CSSCD	143
		SMAD3	15q22.33	4088	rs10518707	0.056	CSSCD	143
					**rs8038623**	0.043	CSSCD	143
		KDR	4q11–q12	3791	rs6554233	0.07	CSSCD	143
					rs6828477	0.066	CSSCD	143
					rs7654599	0.063	CSSCD	143
					**rs2305948**	0.062	CSSCD	143
		MAP3K7	6q16.1–q16.3	6885	rs1145729	0.069	CSSCD	143
					rs157681	0.056	CSSCD	143
		NOX3	6q25.1–q26	50508	rs231944	0.068	CSSCD	143
					**rs231945**	0.066	CSSCD	143
					rs9371889	0.067	CSSCD	143
					**rs6557420**	0.066	CSSCD	143
		NOS3	7q36	4846	**rs1008140**	0.059	CSSCD	143
					rs743507	0.067	CSSCD	143
					rs1808593	0.068	CSSCD	143
		ASS	9q34.1	445	**rs590086**	0.066	CSSCD	143
					rs652313	0.067	CSSCD	143
					rs12555797	0.069	CSSCD	143
					rs543048	0.067	CSSCD	143
		NOS1	12q24.2–q24.31	4842	rs2682820	0.068	CSSCD	143
					rs3825102	0.067	CSSCD	143
					rs1483757	0.066	CSSCD	143
					rs816361	0.045	CSSCD	143
					**rs7977109**	0.029	MSH	48
					rs7309163	0.038	MSH	48
		FLT1	13q12	2321	rs7987291	0.064	MSH	48
					rs2387632	0.067	CSSCD	143
					rs9513097	0.066	CSSCD	143
					rs9508026	0.062	CSSCD	143
					rs8002446	0.067	CSSCD	143
					rs638889	0.065	CSSCD	143
					rs2256849	0.066	CSSCD	143
					rs670084	0.067	CSSCD	143
					rs600640	0.065	CSSCD	143
					rs9319428	0.047	MSH	48
					**rs2182008**	0.003	MSH	48
					rs8002446	0.011	MSH	48
					rs3751395	0.039	MSH	48
					rs9319428	0.047	MSH	48
					rs2387634	0.037	MSH	48
		ALOX5AP	13q12	241	rs4468448	0.064	CSSCD	143
					**rs4769058**	0.057	CSSCD	143
					rs12019512	0.069	CSSCD	143
					rs4445746	0.068	CSSCD	143
		KL	13q12	9365	rs398655	0.062	CSSCD	143
					rs577912	0.068	CSSCD	143
					rs7982726	0.069	CSSCD	143
					rs685417	0.065	CSSCD	143
					**rs9527025**	0.050	CSSCD	143
					rs648202	0.067	CSSCD	143
		PDE7B	6q23-q24	27115	rs2327669	0.041	MSH	48
					rs11154849	0.05	MSH	48
					rs9376173	0.049	MSH	48
					rs1480642	0.044	MSH	48
					**rs487278**	0.017	MSH	48
		HAO2	1p13.3–p13.1	51179	**rs10494225**	0.0039	MSH	48
		MAP3K5	6q22.33	4217	rs9376230	0.036	MSH	48
					rs9483947	0.034	MSH	48
					**rs2237262**	0.03	MSH	29
		ARG2	14q24.1–q24.3	384	rs10483801	0.0075	MSH	48
					**rs10483802**	0.0038	MSH	48
		NOS2A	17q11.2–q12	4843	rs1137933	0.031	MSH	48
					**rs944725**	0.02	MSH	48
		HBS1L	6q23–q24	10767	rs52090901	1.10-5	North European	46
					**rs9399137**	1.10-45	North European	46
					rs4895440	0.00031	North European	46
		PDE7B	6q23–q24	27115	**rs509342**	0.03	MSH	29
		BTF	6q22–q23	9774	**rs703193**	0.05	MSH	29
		MAP7	6q23.3	9053	**rs2179288**	0.003	MSH	29
					rs2076192	0.04	MSH	29
					rs9977139	0.03	MSH	29
					rs3778314	0.02	MSH	29
		PEX7	6q21–q22.2	5191	rs2012700	0.05	MSH	29
					**rs3799476**	0.01	MSH	29
					**rs1342645**	0.01	MSH	29
		Unknown	6q22	N/S	**rs1342641**	0.004	MSH	29
		Unknown	6q22	N/S	**rs44450**	0.01	MSH	29
		BCL11A	2p16.1	53335	rs11886868	10^−20^	Sardinia	144
					rs11886868	4.10^−35^	CCSCD, Brazil	142
					**rs4671393**	2.10^−42^	CCSCD, Brazil	142
					rs7557939	6.10^−38^	CCSCD, Brazil	142
		HBS1L-MYB	6q22-q24	10767–4602	rs28384513	0.04	CCSCD, Brazil	142
					rs7776054	0.0009	CCSCD, Brazil	142
					**rs9399137,**	5. 10^−11^	CCSCD, Brazil	142
					rs9389268	0.0002	CCSCD, Brazil	142
					rs4895441	1.10^−7^	CCSCD, Brazil	142
					**rs6929404**	0.0002	North European	46
**Stroke**	**Large vessel**	HLA-A (*)	6p21.3	3105	0102	0.02	CSSCD	60
					**2612**	0.007	CSSCD	60
					**3301**	0.04	CSSCD	60
		IL-4R	16p12.1–p11.2	3566	**rs1805015, S503P**	0.006	CSSCD, STOP	61, 63
		TNFa (*)	6p21.3	7124	**rs1800629, −308G**>**A**	0.048	CSSCD, STOP	61, 63
		ADRB2 (*)	5q31–q32	154	**rs1042714, Q27E**	0.033	CSSCD	145
**Stroke**	**Small vessel**	HLA DPB1 (*)	6p21	3115	**0401**	0.01	CSSCD	60
					**1701**	0.02	CSSCD	60
		VCAM1	1p32–p31	7412	**rs1041163, −1594T**>**C**	0.002	CSSCD	145
		LDLR	19p13.3	3949	**rs5742911**	0.002	CSSCD	145
**Stroke**	**Vessel size N/S**	ADCY9	16p13.3	115	N/S	N/S	N/S	59
					rs437115	a3	CSSCD	66
					rs2238432	a98	CSSCD	66
					**rs2238426**	a3381	CSSCD	66
					rs2072338	a638	CSSCD	66
					rs2283497	a10	CSSCD	66
		ANXA2	15q22.2	302	**hCV26910500**	a1.68 10^8^	CSSCD	66
		BMP6	6p24.3	654	rs267196	a2.31 10^16^	CSSCD	66
					**rs267201**	a1.92 10^103^	CSSCD	66
					rs408505	a4.06 10^101^	CSSCD	66
					rs449853	a2.20 10^57^	CSSCD	66
		CCL2	17q11.2–q12	6347	**rs4586**	a844	CSSCD	66
		CSF2	5q31.1	1437	**rs25882**	a1.19 10^198^	CSSCD	66
		ECE1	1p36.12	1889	rs212528	a1.55 10^4^	CSSCD	66
					**rs212531**	a2.34 10^80^	CSSCD	66
		ERG	21q22.3	2078	**rs989554**	a62	CSSCD	66
		MET	1q31.1	4233	rs38850	a68	CSSCD	66
					**rs38859**	a1.58 10^39^	CSSCD	66
		SELP	1q24.2	6403	rs2420378	a1.90 10^10^	CSSCD	66
					**rs3917733**	a2.84 10^93^	CSSCD	66
					rs3753306	a2.32 10^65^	CSSCD	66
		TEK	9p21	7010	**rs489347**	a2	CSSCD	66
		TGFBR3	1p22.1	7049	N/S	N/S	N/S	59
					**rs284875**	a443992	CSSCD	66
					rs2148322	a68988	CSSCD	66
					rs2765888	a41968	CSSCD	66
					rs2007686	a1739	CSSCD	66
		HLA DRB1	6p21.3	3123	**0301**	0.007	African American	146
		(*)			**0302**	0.007	African American	146
					**1501**	0.019	African American	146
		HLA DQB1	6p21.3	3119	**0201**	0.033	African American	146
		(*)			**0602**	0.011	African American	146
		VCAM1 (*)	1p32–p31	7412	**rs3783613, G1238C**	0.04	Jamaican, STOP	4, 64
		AGT	1p42–q43	183	**GT repeat**	0.05	African American	147
**Avascular Necrosis**		MTHFR	1p36.3	4524	**C677T**	0.006	N/S	76
		BMP6	6p24–p23	654	rs270393	0.009	CSSCD	81
					**rs267196**	0.001	CSSCD	81
					rs267201	0.008	CSSCD	81
					rs449853	0.012	CSSCD	81
					**rs1225934**	0.001	CSSCD	81
		TGFBR2	3p22	7048	rs1019856	0.023	CSSCD	81
					**rs934328**	<0.001	CSSCD	81
		TGFBR3	1p22.1	7049	**rs284157**	<0.001	CSSCD	81
		EDN1	6p24.1	1906	**rs5369**	0.001	CSSCD	81
					**hCV7464888**	0.001	CSSCD	81
		ERG	21q22.3	2078	rs979091	0.014	CSSCD	81
					**rs2836430**	0.005	CSSCD	81
		KL	13q12	9365	**rs480780**	0.001	CSSCD	81
					**rs211235**	0.001	CSSCD	81
					rs2149860	0.021	CSSCD	81
					rs685417	0.002	CSSCD	81
					rs516306	0.019	CSSCD	81
					**rs565587**	0.001	CSSCD	81
					**rs211239**	0.001	CSSCD	81
					**rs211234**	0.001	CSSCD	81
					rs2238166	0.046	CSSCD	81
					rs499091	0.020	CSSCD	81
					rs576404	0.010	CSSCD	81
		ECE1	1p36.12	1889	**rs212527**	<0.001	CSSCD	81
		ANXA2	15q22.2	302	rs7163836	0.010	CSSCD	81
					**hCV11770326**	<0.001	CSSCD	81
					**rs7170178**	<0.001	CSSCD	81
					rs1033028	0.007	CSSCD	81
					**hCV26910500**	<0.001	CSSCD	81
					hCV1571628	0.034	CSSCD	81
		STARD13	13q12–q13	90627	rs538874	0.015	CSSCD	81
					rs475303	0.029	CSSCD	81
					**rs648464**	0.001	CSSCD	81
		APRIN	13q12.3	23047	**hCV3118898**	0.001	CSSCD	81
					hCV11710292	0.014	CSSCD	81
**Acute Chest Syndrome**		NOS3	7q36	4846	**T-786C**	0.0051	African American	82
						0.021	Guadeloupe	85
		NOS1	12q24.2–q24.31	4842	**(AAT)n intron 20**	0.005	African American	84
		ET-1	6p24.1	1906	**T8002C**	0.039	Guadeloupe	85
**Priapism**		KL	13q12	9365	**rs2249358**	b2.6	CSSCD	87
					rs211239	b1.7	CSSCD	87
					rs211234	b2.3	CSSCD	87
		TGFBR3	1p22.1	7049	**rs7526590**	0.00058	N/S	88
		AQP1	7p14	358	**rs10244884**	0.00068	N/S	88
		ITGAV	2q31–q32	3685	**rs3768780**	0.00090	N/S	88
		F13A1	6p25.3–p24.3	2162	**hcv1860621**	0.00156	N/S	88
**Leg Ulcers**		TGFBR3	1p22.1	7049	**rs2038931**	0.0387	CSSCD	148
		TGFBR2	3p22	7048	**rs1019856**	0.0170	CSSCD	148
		BMP6	6p24–p23	654	**rs270393**	0.0362	CSSCD	148
		TEK	9p21	7010	**rs603085**	0.0108	CSSCD	148
					rs671084	0.0251	CSSCD	148
		KL	13q12	9365	rs685417	0.0186	CSSCD	148
					**rs516306**	0.0076	CSSCD	148
					rs2149860	0.0480	CSSCD	148
		APRIN	13q12.3	23047	**hCV3118898**	0.0153	CSSCD	148
		BMPR1B	4q22–q24	658	**rs1560909**	0.0263	CSSCD	148
					rs7661539	0.0389	CSSCD	148
		MAP3K7	6q16.1–q16.3	6885	**rs157702**	0.0080	CSSCD	148
		SMURF1	7q22.1	57154	**rs219825**	0.0389	CSSCD	148
		SMAD9	13q12–q14	4093	**rs9576135**	0.0167	CSSCD	148
		Unknown	18q21.1	N/S	**rs736839**	0.0004	CSSCD	148
		MAP2K1	15q22.1–q22.33	5604	**rs8036023**	0.0414	CSSCD	148
**Gallstone**		UGT1A	2q37	7361	**Promoter (TA)n**	0.0015	see text	106
**Infection and Bacteriamia**		MPO	17q23.1	4353	**G463A**	0.0112	Brazilian	111
		IGF1R	15q26.3	3480	**rs1319868**	0.0059	CSSCD	108
					rs1567811	0.0586	CSSCD	108
					rs8041224	0.0133	CSSCD	108
					rs2872060	0.0321	CSSCD	108
		BMP6	6p24–p23	654	rs270387	0.0401	CSSCD	108
					rs267188	0.0047	CSSCD	108
					**rs408505**	0.0006	CSSCD	108
					rs449853	0.02	CSSCD	108
		TGFBR3	1p22.1	7049	**rs2765888**	0.0251	CSSCD	108
		BMPR1A	10q22.3	657	rs6586039	0.0248	CSSCD	108
					**hCV1663921**	0.0115	CSSCD	108
		SMAD6	15q21–q22	4091	**rs5014202**	0.0324	CSSCD	108
		SMAD3	15q22.33	4088	**rs10518707**	0.0114	CSSCD	108
		Unknown	1p22.1	N/S	**rs6662385**	0.0222	CSSCD	108
		HLA-E	6p21.3	3133	**0101**	0.003	African	110
**Pulmonary Hypertension**		ACVRLI	12q11–q14	94	**rs3847859**	0.003	African American	115
					rs706814	0.009	African American	115
		ADCY6	12q12–q13	112	**rs9804777**	0.017	African American	115
		ADRB1	10q24–q26	153	**rs1801253**	0.006	African American	115
		BMP6	6p24–p23	654	rs267192	0.014	African American	115
					**rs267196**	0.013	African American	115
					rs267201	0.019	African American	115
		BMPR2	2q33–q34	659	**rs17199249**	0.018	African American	115
					rs35711585	0.025	African American	115
		CR1	1q32	1378	**rs6663530**	0.024	African American	115
		FY	1q21–q22	2532	**rs3027045**	0.035	African American	115
		KL	13q12	9365	**rs1888057**	0.031	African American	115
		LCAT	16q22.1	3931	rs5923	0.033	African American	115
					**hcv2846928**	0.014	African American	115
		LTA4H	12q22	4048	**rs10492226**	<0.001	African American	115
					rs1978331	0.022	African American	115
		SELP	1q22–q25	6403	**rs2235302**	0.014	African American	115
					rs6131	0.019	African American	115
		SERPINCI	1q23–q25.1	462	**rs2227617**	0.001	African American	115
		SLC12A6	15q13	9990	**rs426634**	0.028	African American	115
		TGFBR3	1p22.1	7049	**rs10874940**	0.002	African American	115
					rs17443164	0.045	African American	115
**Glomerular**		BMPR1B	4q22–q24	658	rs2240036	0.0434	CSSCD	94
**Filtration**					rs4145993	0.0352	CSSCD	94
**Rate**					rs17022863	0.011	CSSCD	94
					**rs1434549**	0.0109	CSSCD	94

**Abbrevations:** CSSCD, Cooperative Study of Sickle Cell Disease; MSH, Multicenter Study of Hydroxyurea in SCA; STOP, Stroke Prevention Trial in Sickle Cell Anemia study.

**Notes:**

a Bayes factor of the model associating the SNP to stroke versus the model of independence (large Bayes factor implies that there is very strong evidence for the associations).^66^

b Odd ratio.^87^ In **Bold** is the highest significance SNP for each gene.
